# Supplementary material for: Antisense oligonucleotide-mediated Dnm2 knockdown prevents and reverts myotubular myopathy in mice
Source: Nat Commun. 2017 Jun 7;8:15661. doi: 10.1038/ncomms15661 (PMC5467247; doi:10.1038/ncomms15661)
Supplement: Supplementary Information — Supplementary figures and supplementary tables. [file ncomms15661-s1.pdf]

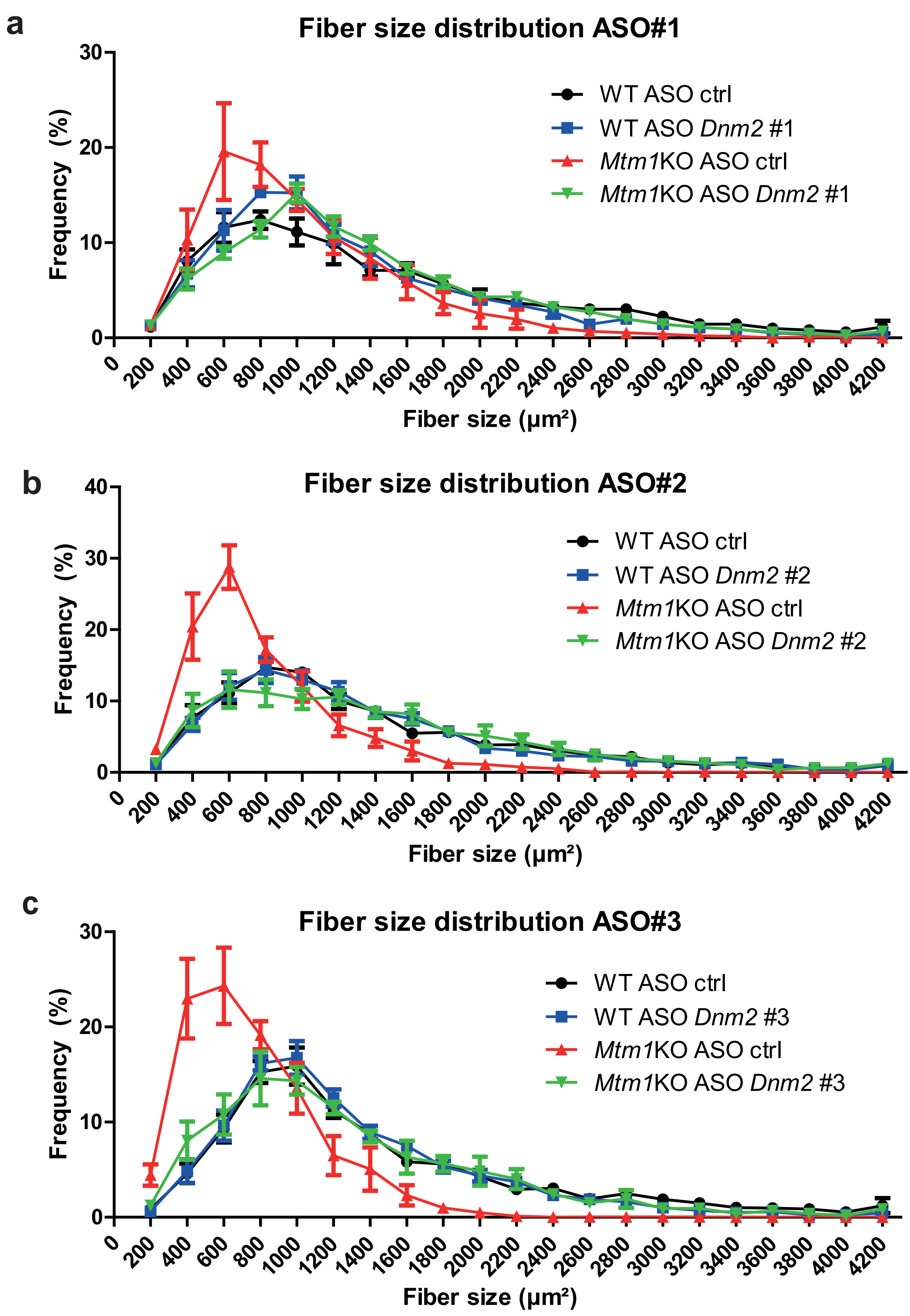

Supplementary Figure 1: Fiber size distribution of WT or *Mtm1*KO TA muscles injected with ASOs according to their size. Myofiber area was measured on 8 µm sections of TA muscles that were injected with ASO#1 (**a**), #2 (**b**) , #3 (**c**) or contralateral TA injected with ASO control. They have been subdivided into 21 subgroups ranging from the smallest to the largest. The curve represents the percentage of fibers that belongs to each subgroup. >300 fibers were quantified per sample, *n*=5 per group.

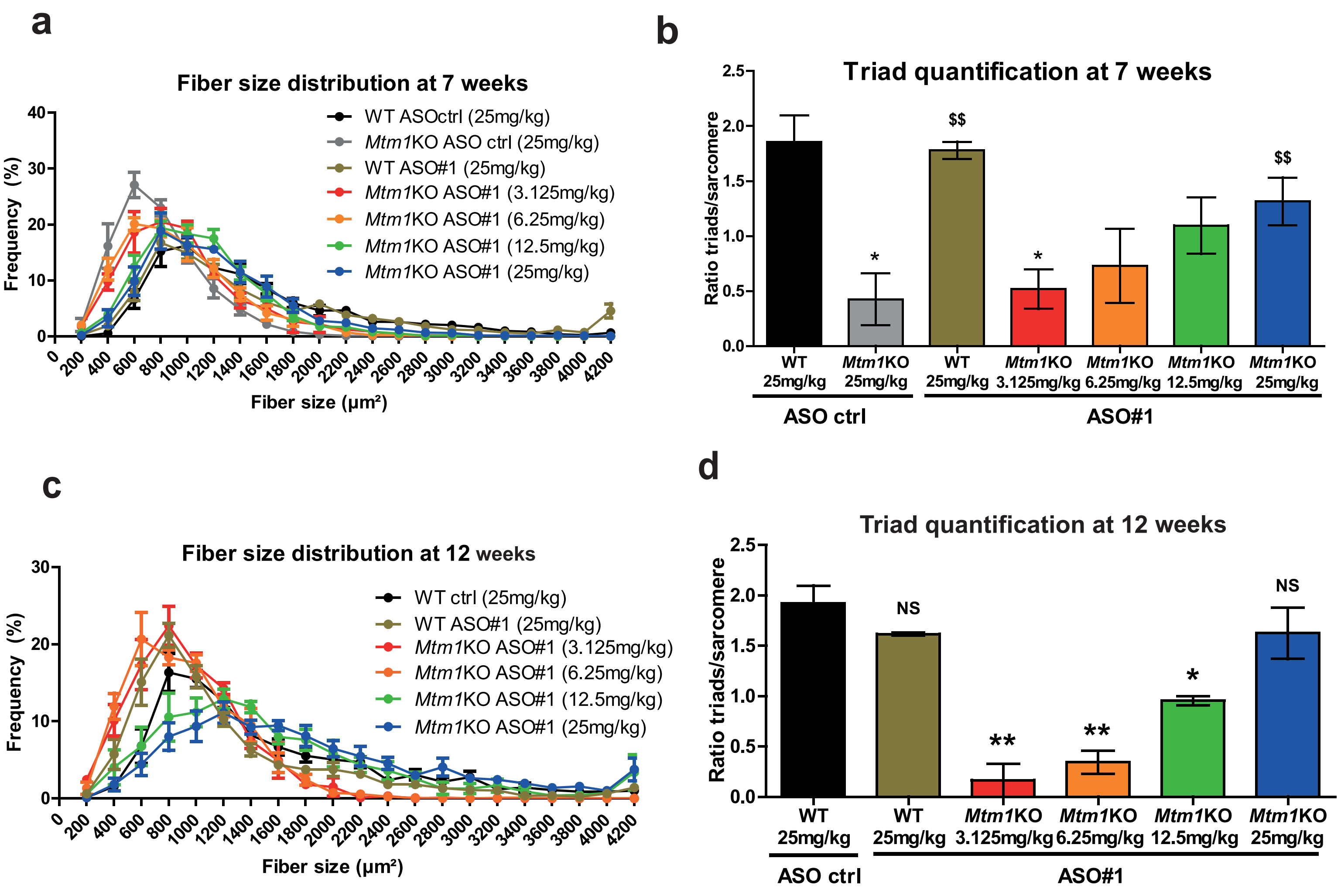

Supplementary Figure 2: Muscle organisation and histology evaluation after systemic ASO#1 treatment for 5 or 11 weeks. (**a**) and (**c**) myofiber area was measured on 8 µm TA muscle sections. The curve represents the percentage of fibers that belong to each subgroups. >300 fibers were quantified per group, *n*=5. (**b**) and (**d**) triads/sarcomere ratio was determined by dividing the number of triads observed by the number of sarcomeres present in the field, *n*=3. Data represent means ± SEM. *NS*: no statistical significance, *p<0.05, **p<0.01 vs WT treated with ASO ctrl. $$$p$<0.01 vs *Mtm1*KO treated with ASO ctrl (ANOVA test followed by post-hoc Bonferroni).

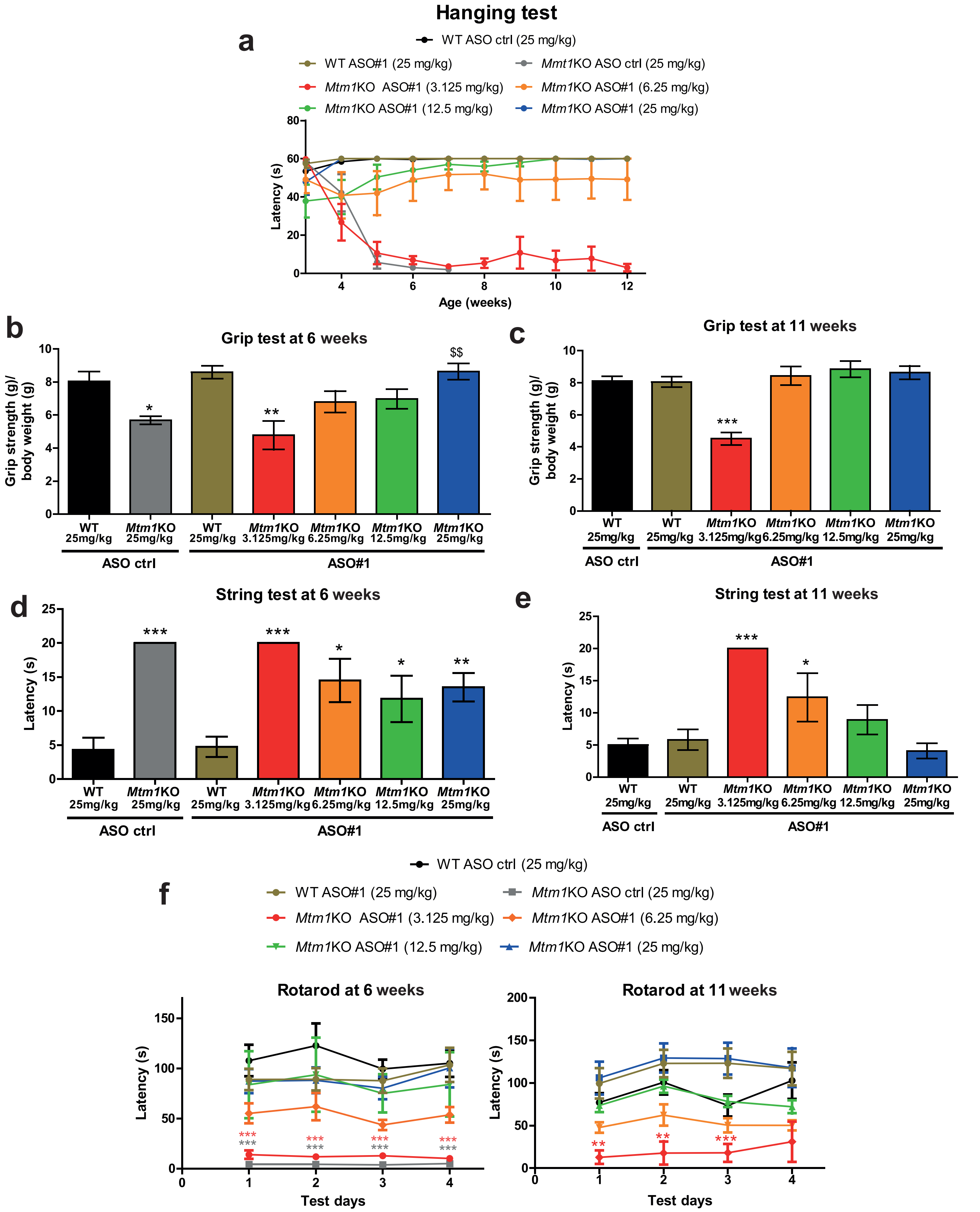

Supplementary Figure 3: Clinical improvement of the CNM phenotype after 5 or 10 weeks of systemic ASO treatment. (**a**) The ability of WT or *Mtm1*KO mice to perform the hanging test was measured every week. Quantification of mice muscle strength, fine motor coordination, balance and resistance to fatigue were assessed with grip (**b**, **c**), string (**d**, **e**) and rotarod (**f**), at week 6 (**b**, **d**, **f**) and week 11 (**c**, **e**, **f**). *n*=6-7 except for *Mtm1*KO treated with ASO#1 at 3.125 mg/kg at 11 weeks where *n*=3. Data represent means ± SEM. *p<0.05, **p<0.01, ***p<0.001 for mice treated with ASO#1 vs WT treated with ASO ctrl. $$$p$<0.01 for *Mtm1*KO treated with ASO#1 vs *Mtm1*KO treated with ASO ctrl (ANOVA test followed by post-hoc Bonferroni).

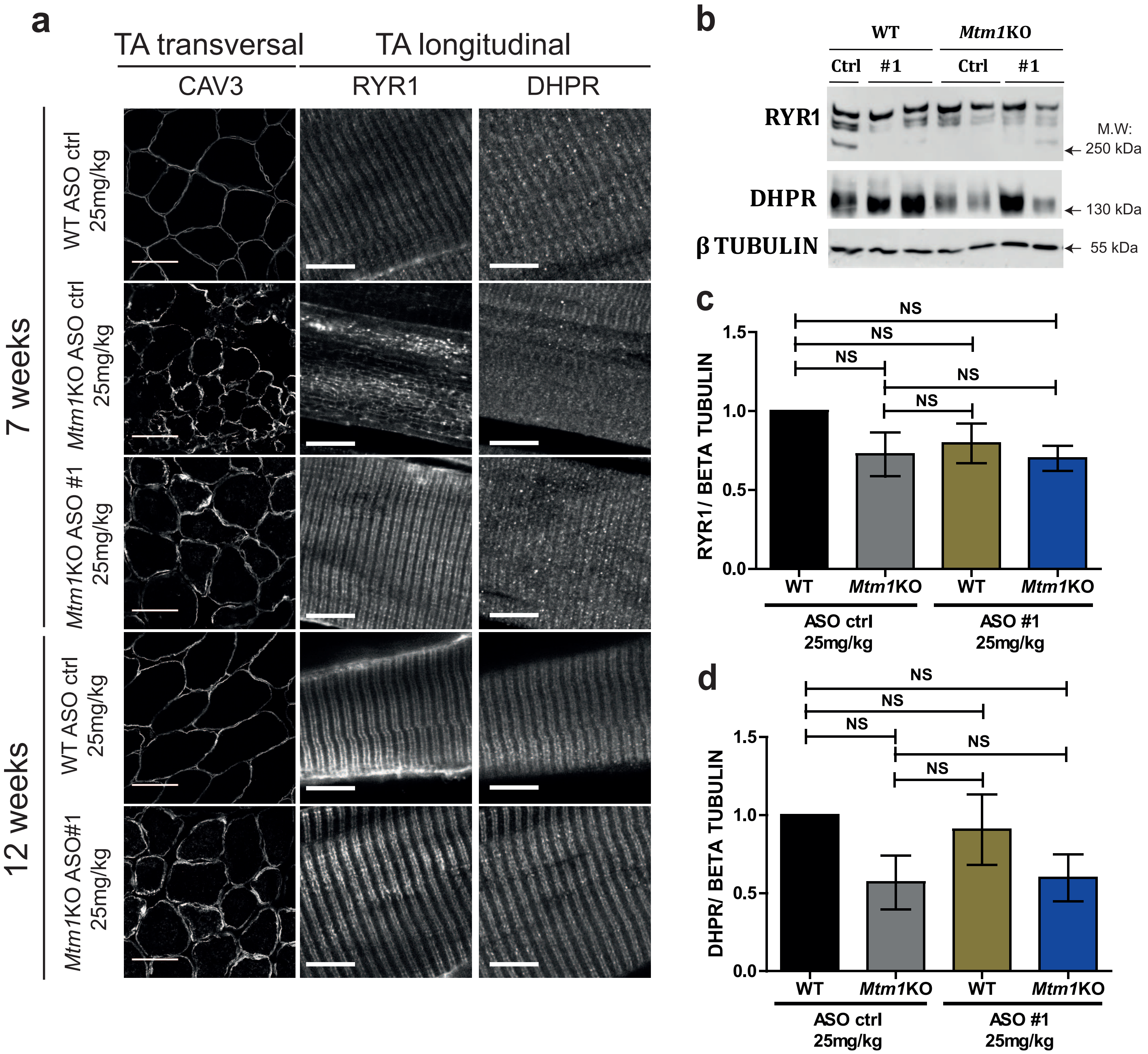

Supplementary figure 4: RYR1 and DHPR localization expression in TA muscle of WT or *Mtm1*KO mice treated with systemic ASO ctrl or ASO#1 injection. (**a**) Immunostaining of CAV3, RYR1 or DHPR in tibialis anterior muscle sections of WT or *Mtm1*KO mice treated with ASO ctrl or ASO#1 at 7 weeks or 12 weeks. CAV3 localisation was assessed on transversal sections (scale bars: 50µm) while RYR1 and DHPR were localized on longitudinal sections (scale bars: 10µm). (**b**) Western blot of RYR1, DHPR and Beta TUBULIN (loading control) of TA muscle lysates at 7 weeks. Relative level of RYR1 (**c**) and DHPR (**d**) levels were determined by densitometry of RYR1 or DHPR standardized to Beta TUBULIN. *n*=4. Data are means ± SEM. *NS*: no statistical significance. (ANOVA test followed by post-hoc Bonferroni). M.W: molecular weight. kDa: kilodalton.

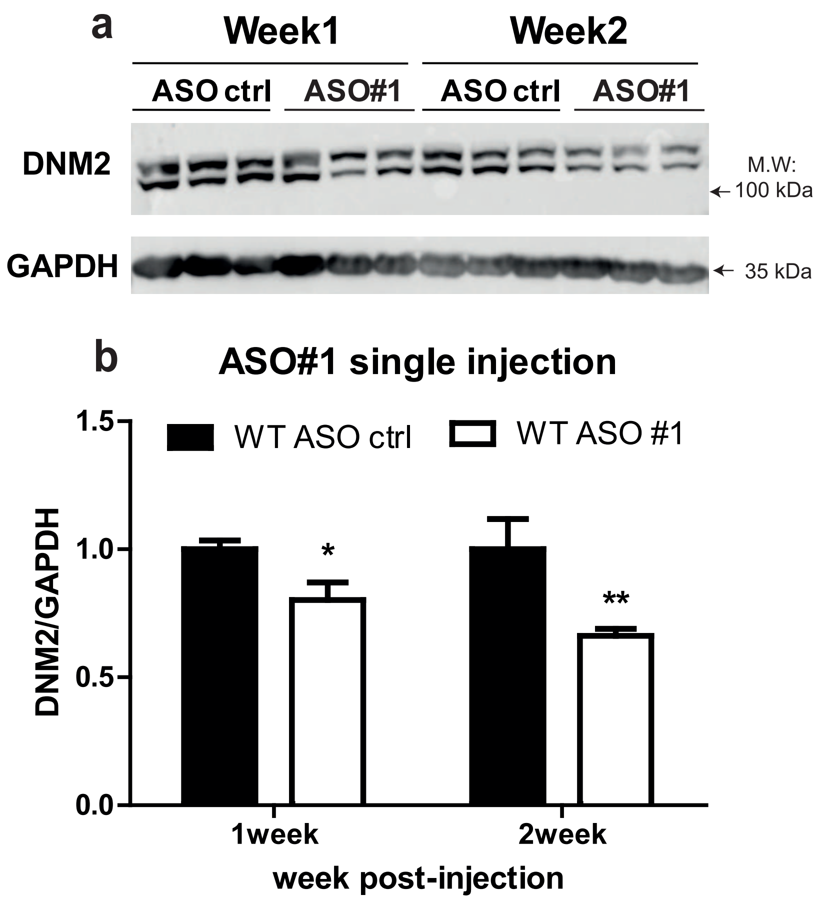

Supplementary Figure 5: DNM2 downregulation persistence after a single injection at week 3. (**a**) Blots represent DNM2 protein level in WT TA muscle lysates 1 or 2 weeks after a single intraperitoneal injection of 25 mg/kg of ASO#1. (**b**) Graph depicts the densitometry quantification of DNM2 level relative to GAPDH (loading control). n=3. Data are means ± SEM. *p<0.05, **p<0.01 (t-test). M.W: molecular weight. kDa: kilodalton.

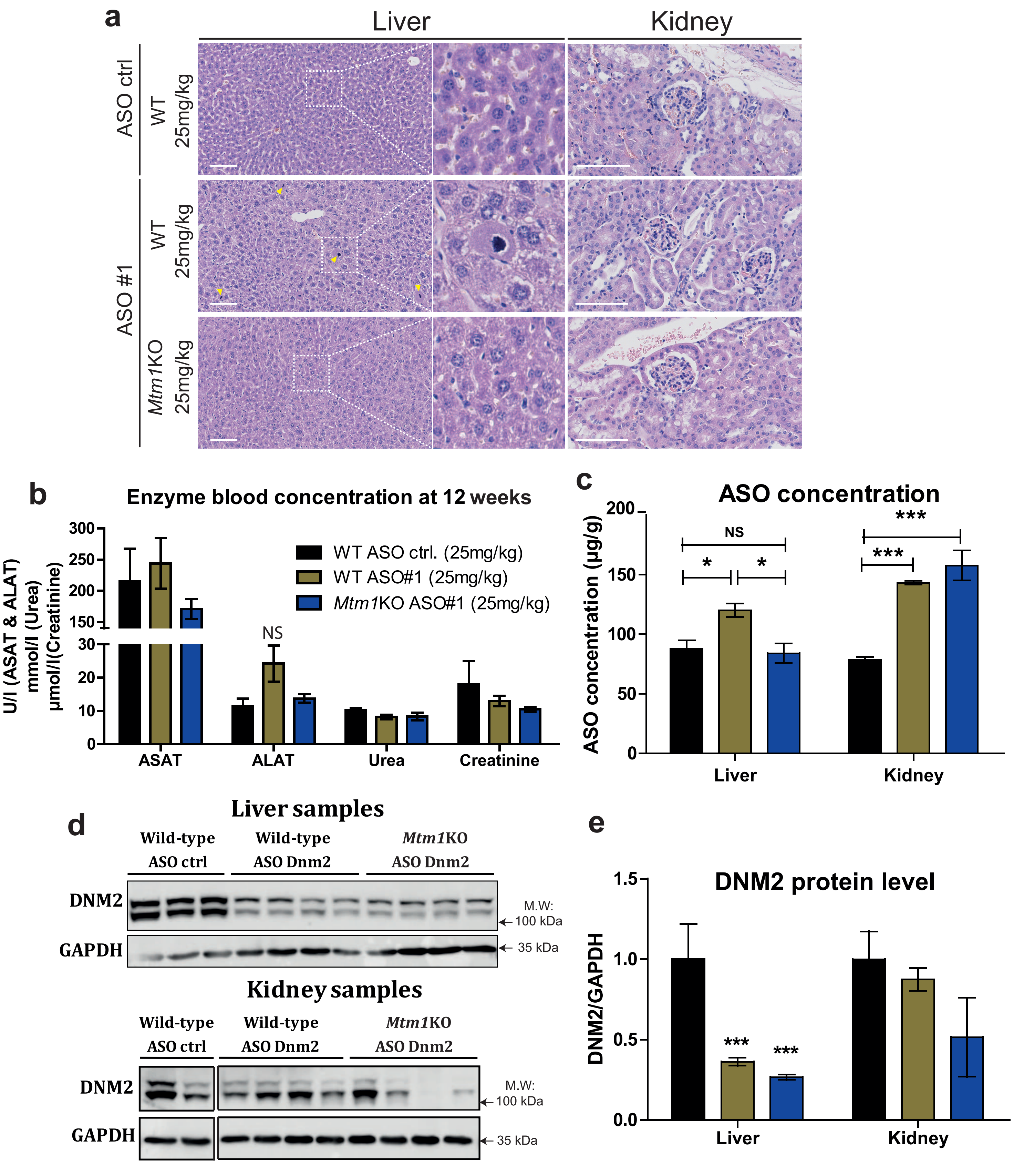

Supplementary figure 6: Systemic ASO treatment effects on livers and kidneys of WT mice treated with ASO ctrl or ASO#1 or *Mtm1*KO mice treated with ASO#1 at 25 mg/kg for 10 weeks. (**a**) H&E staining of liver and kidney sections. Yellow arrows show some hepatocyte with cytoplasm enlargement and condensed nuclear material found in WT treated with ASO#1. The zoom magnified examples of hepatocytes observed in the analyzed samples. Scale Bars: 100µm. (**b**) Liver and kidney serum biomarkers were analyzed at age of 12 weeks. Graphs represent ASAT, ALAT, urea and creatinine blood levels. (**c**) ASO concentration in liver or kidney homogenates. ASO concentration was determined using mass spectrometry. (**d**) Blots show DNM2 and GAPDH (loading control) expression in liver and kidney samples. (**e**) Graphs representing the quantification of DNM2/GAPDH ratio. *n*= 3-4 per group. Data are means ± SEM. *NS*: no statistical significance. **p*<0.05, ****p*<0.001 (ANOVA test followed by post-hoc Bonferroni). M.W: molecular weight. kDa: kilodalton.

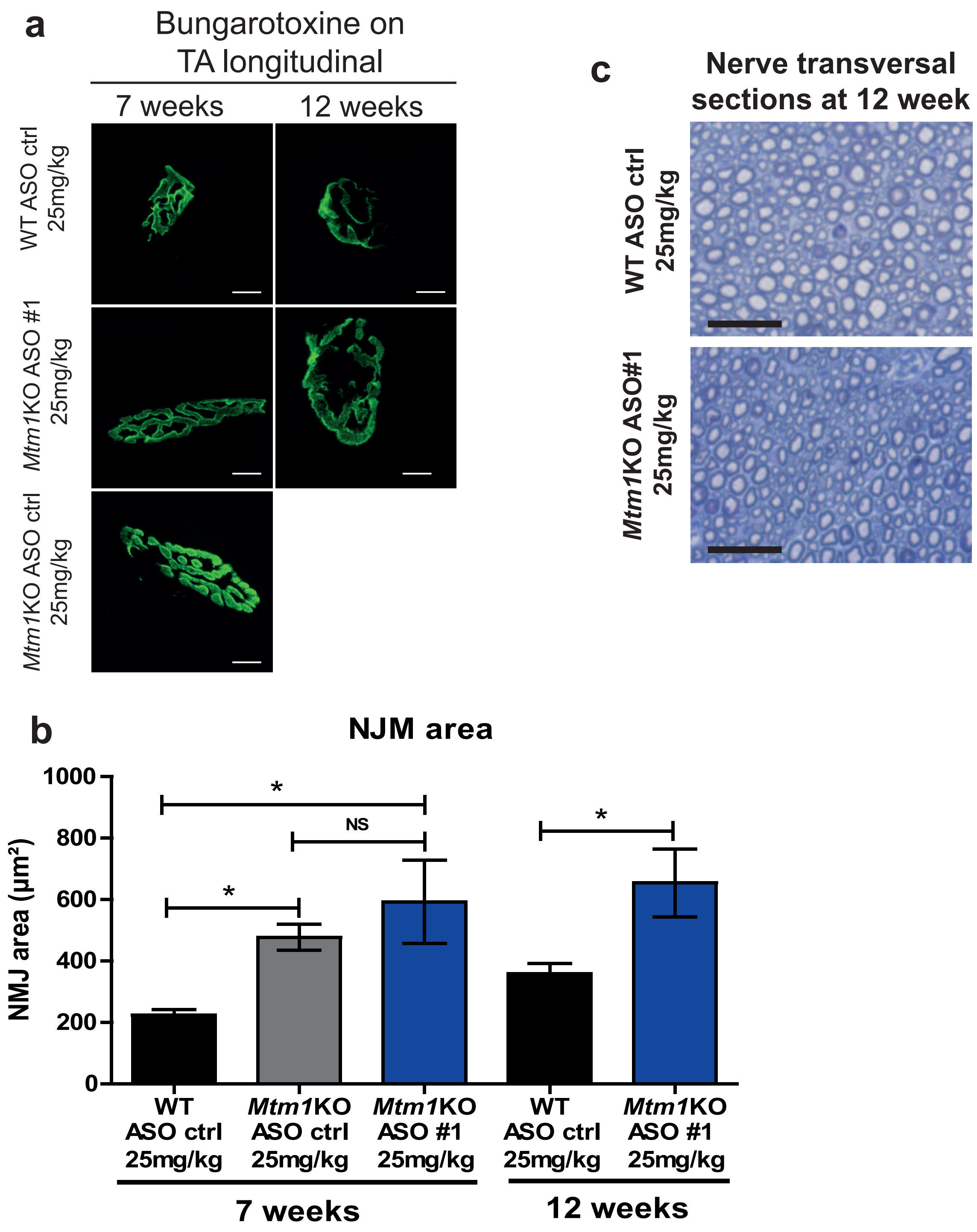

Supplementary Figure 7: Neuromuscular and peripheral nerve assessment. (**a**) Neuromuscular junctions were analysed on longitudinal sections using CF™488A α-bungarotoxin. Scale bars: 10 µm. (**b**) Graphs representing a quantification mean ± SEM of the NMJ overall area. *n*=5-6 per group . *NS*: not statistically significant, **p*<0.05 vs age-matched WT ASO ctrl (ANOVA test followed by post-hoc Bonferroni). (**c**) Toluidine blue staining of sciatic nerve transversal sections. Scale bars: 25 µm.

Figure 1c

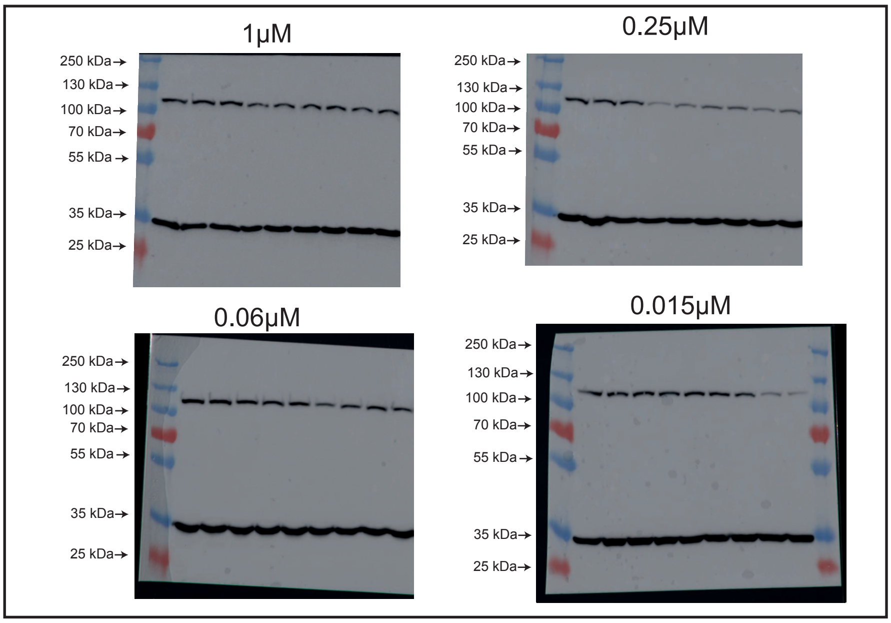

Figure 2a

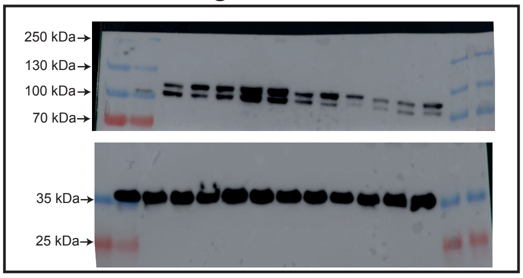

Figure 7a

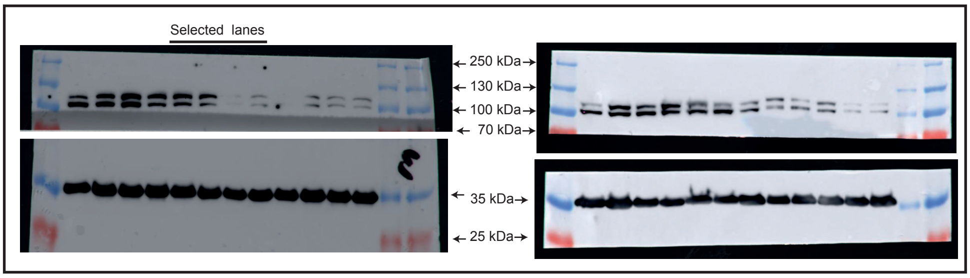

Figure 7b

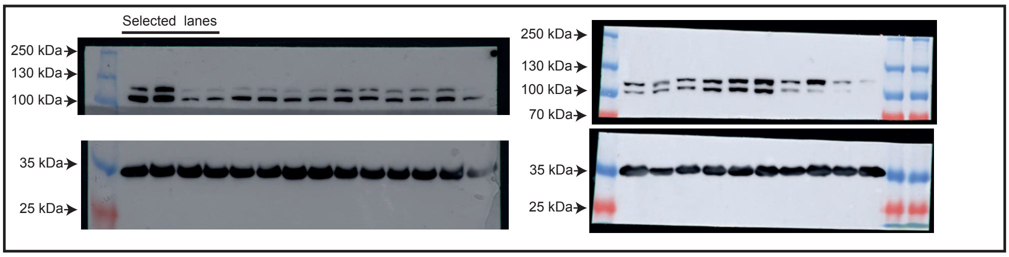

Figure 9h

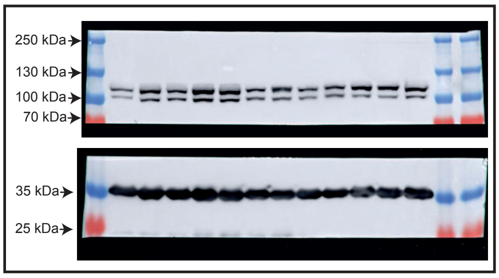

Supplementary Figure 4b

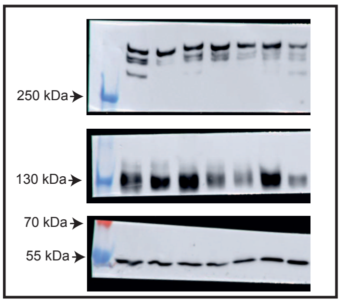

Supplementary Figure 5a

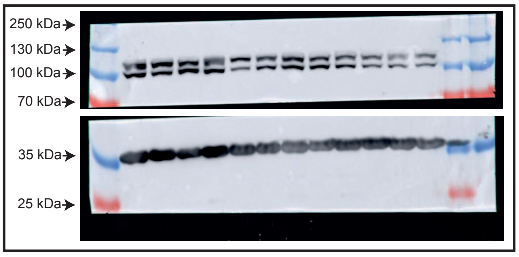

Supplementary Figure 6d

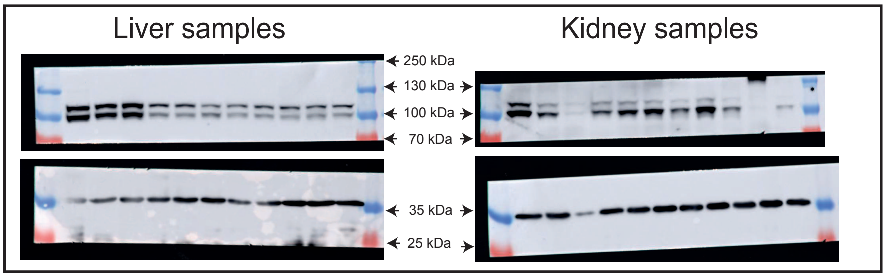

Supplementary Figure 8: Uncropped western blots used in figures 1c, 2a, 7a, 7b, 9h and supplementary figures 4b, 5a, 6d
Full western blot with marker size indicated by the arrows. kDa: kilodalton

ASO No (IONIS ID)	Length	Sequence (5’-3’)
ASO ctrl. (549144)	16	**GGC**CAATACGCCG**TCA**
ASO #1 (694966)	16	**GGC**ATAAGGTCAC**GGA**
ASO #2 (695233)	16	**CGC**AGGAACCCCC**TCA**
ASO#3 (695244)	16	**GGT**AGACCCCAGC**ACG**

Unmodified DNA

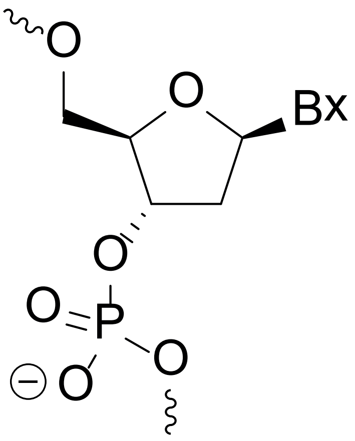

phosphorothioate constrained ethyl (cEt) ASO

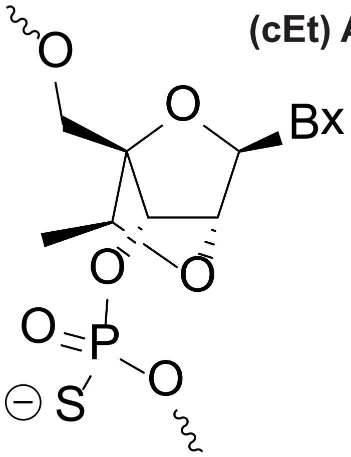

Supplementary Table 1: sequence (top) and representation of the chemical structure (bellow) of antisense oligonucleotides (ASOs). All ASO have phosphorothioate backbone and constrained ethyl (cET) modifications on the wings (**bold**) with a deoxy gap (underlined).

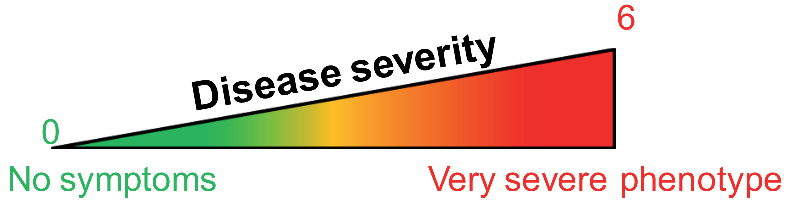

Score =	0	0,5	1	
Diff. of body weight (compared to WT littermates)	0 g	1-2g	> 2g	0-1
Hanging test ability	60s	5-60s	<5s	0-1
Walking (hindlimbs)	Normal	Splayed	Loss of use	0-1
Ptosis	No	X	Yes	0-1
Kyphosis	No	Mild	Severe	0-1
Breathing alteration	No	x	Yes	0-1
Total: **D**isease **S**everity **S**core (**DSS**)				**= 0-6**

Supplementary Table 2: The six CNM features used to score the disease severity (DSS) in *Mtm1*KO mice. *Mtm1*KO mice were followed every week for the six CNM features (before and after ASO treatment). A score of 0 to 1 was given for each feature and the sum represents the DSS. The higher is the DSS, more severe is the phenotype.
